# Preparation, characterisation and microbiological examination of Pickering nano-emulsions containing essential oils, and their effect on Streptococcus mutans biofilm treatment

**DOI:** 10.1038/s41598-019-52998-6

**Published:** 2019-11-12

**Authors:** Barbara Horváth, Viktória L. Balázs, Adorján Varga, Andrea Böszörményi, Béla Kocsis, Györgyi Horváth, Aleksandar Széchenyi

**Affiliations:** 1 Institue of Pharmaceutical Technology and Biopharmacy, Faculty of Pharmacy, University of Pécs, Rókus str. 2., H-7624 Pécs, Hungary; 2 Department of Pharmacognosy, Faculty of Pharmacy, University of Pécs, Rókus str. 2, H-7624 Pécs, Hungary; 3 Department of Medical Microbiology and Immunology, Faculty of Medicine, University of Pécs, Szigeti str. 12, H-7624 Pécs, Hungary; 4 Department of Pharmacognosy, Faculty of Pharmacy Semmelweis University, Üllői str. 26, H-1085 Budapest, Hungary

**Keywords:** Microbiology, Drug regulation

## Abstract

Essential oils (EOs) are commonly applied in mouth care products like mouthwashes, mostly as an ethanolic solution or by usage of surfactants as solubilising agents. In this study, we present a formulation for preparation of Pickering nano-emulsions (PnE) of EOs as a novel form for application of EOs in mouth care. For the preparation of PnE, we have synthesised surface-modified silica nanoparticles with a mean diameter of 20 nm, as well as we have examined the effect of EOs concentration on PnE droplet size and stability. *In vitro* study of their effect on the *Streptococcus mutans* biofilm as the main pathogen of dental health problems has been performed. We have found that EOs in the PnE form has the highest effectiveness against biofilm formation. Diffusion through the biofilm model membrane was studied to explain this observation. We have found that PnEs have a better performance in the transportation of EOs trough model membrane than the ethanolic solutions and conventional emulsions (CEs).

## Introduction

Dental plaque is a thin biofilm layer built by microorganisms, mainly Streptococcus species^1^. Microbial activity in the dental plaque causes a local decrease of pH value and weakens the mineralised tooth structures that can lead to several tooth diseases like caries, gingivitis, and periodontitis. For this reason, the removal of dental plaque is the most important part of mouth hygiene. The daily removal is commonly achieved by mechanical methods, such as tooth brushing, usage of tooth floss or interdental brushing^2^. Tooth brushing is used by most of the population, while only a small percentage uses tooth floss or interdental brushing regularly^3^. The efficacy of mechanical methods differs in a great extent. The plaque removal by tooth brushing mainly depends on the time and technique of brushing, and on the quality of toothbrushes^4^, but usually, the desired plaque removal is not reached by this method. That is why an additional technique, such as chemical plaque removal or prevention, is often used. Chemical plaque removal or prevention can be achieved by application of mouthwashes, whom usage is widespread among the grown population. The commercially available mouthwashes usually contain aminefluoridine, chlorhexidine, hexetidine, octenidine, triclosan or plant extracts as antibacterial agents^5^. Most of them cause some side effect after prolonged usage, except plant extracts like EOs. G. Pizzo *et al*. demonstrated that EOs have the same efficacy as aminefluoridin or CHX so that they can replace these ingredients in mouthwashes^6^.

The EOs based commercial products that are used for treatment or prevention of dental diseases are containing surfactants, solvents or co-solvents to enhance the water solubility of EOs^7^. Among the solvents, different alcohols (e.g. ethanol, propylene glycol, glycerine) are applied in mouthwash or gargles. Commonly used surfactants are polysorbates (20, 60 or 80)^8^ and SLS (Na-lauryl-sulphate)^9^, which are used as emulsion stabilisers or EO solubilising agents. Unfortunately, the surfactants can cause mucous membrane irritation by damaging their cell membranes; additionally, at long-term usage, they can get into the blood circulation and cause other side effects^10^. The alcohols can cause dehydration of the mouth, which makes the mucous membrane more sensitive to infections or other diseases^11^.

Additionally, the application of solvents and surfactants in microbiological tests can lead to misinterpretation of the experimental data, because alcohols and surfactants also have antimicrobial activity^12^ that is why the effect of essential oils cannot be unambiguously determined. To avoid the use of solvents and surfactants, yet to provide the availability of EOs on the test or treatment site, Pickering emulsions can be prepared, which are emulsions stabilised with solid particles^13^. For this purpose, we can use biologically inert, non-toxic particles, e.g. cellulose^14^, silica^15^ or PLGA^16^ particles, which are widely used in pharmaceutical technology.

Pickering emulsions can have the same or better stability than the conventional, surfactant stabilised emulsions interface^17^. The adsorption of the solid particles and surfactants on the liquid-liquid interface is a spontaneous, reversible process. The adsorption energy of solid particles on the liquid-liquid interface is higher than the adsorption energy of surfactants. In some cases, where the adsorption energy of solid particles is extremely high, adsorption process can be considered as irreversible^17^. Because of their stability, Pickering emulsions can be used in pharmaceutical and medical applications.

Beside emulsion stabilisation, the solid particles may interact with biofilm, and targeted EO transportation can also be achieved^18^. The effectiveness of targeted EO delivery depends on the emulsion type, the emulsion stabilisation agent and emulsion droplet size^19,20^. Usually, the biofilm consists of polysaccharide matrix, and it is impermeable for many pharmaceutically active ingredients, that is why the treatment of stiff bacterial colonies is challenging^21^. Because of the hydrophilic properties of *Streptococcus mutans* biofilm^22^, an O/W type emulsion should be applied. When the emulsion is stabilised by the particles with appropriate hydrophilic/lipophilic surface character, which can adsorbed preferably on the biofilm, targeted delivery can be reached. The emulsion droplet size also plays an important role in the delivery of EOs: the mean pore size of biofilms in the top layer ranges from approximately 1.7–2.7 µm and 0.3–0.4 µm in the bottom layers^23^. Because of the pore size of biofilms we can assume, that if the emulsion droplet size is less than 300 nm, the droplets can penetrate deep into the biofilm matrix.

We aimed to prepare O/W type Pickering emulsions with four EOs, such as cinnamon bark (Cinnamomum verum J. Presl.), clove (Syzygium aromaticum (L.) Merr. and Perry), peppermint (Mentha x piperita L.) and thyme EO (Thymus vulgaris L.) and to examine the influence of the EOs concentration on droplet size and stability of Pickering emulsions. Their antimicrobial activities against *Streptococcus mutans* have been studied before^24^, moreover several researchers have found that these EOs can act as biofilm inhibitors with other bacterial strains^25–28^. Their ethanolic solutions and conventional emulsions (CEs) have also been tested to compare the effectiveness of different EO delivery forms.

We have also examined the diffusion properties of different EO forms through model membrane, to explain the difference in their antibacterial or inhibitory effects. In the case of cinnamon EO we have prepared and examined PnE with SNPs that have different hydrophilic/lipophilic surface character to determine its influence on the antibacterial activity and diffusion properties as model membrane agar gel was used, which is a suitable *Streptococcus mutans* biofilm model because they have similar hydrophilic properties and tortuous pore structure^29^.

## Results and Discussion

### Synthesis and characterisation of silica nanoparticles

Hydrophilic silica nanoparticles (HS) were synthesised by a method established by Stöber, Fink and Bohn, the mean diameter was 20 nm, and the PDI was 0.008, determined by DLS. The stability of the PnEs can be influenced by the hydrophilic/lipophilic surface properties of silica nanoparticles^30^. In our previous work^31^, we have ascertained that the PnEs with appropriate stability and tailored size can be prepared with silica nanoparticles, which were partially surface modified with ethyl groups. For this reason, we have modified the surface of HS by ethyl functional groups with a theoretical surface coverage of 20% (20ET) and 40% (40ET).

**Figure 1:**
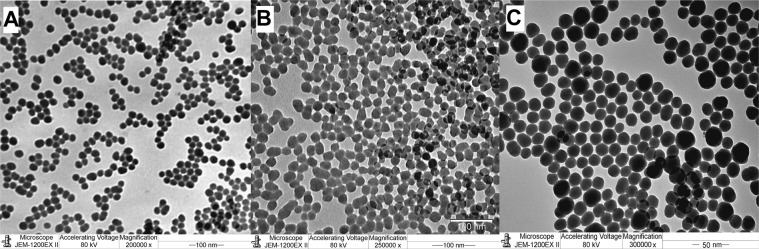
TEM images of silica nanoparticles. (**A**) HS. (**B**) 20ET. (**C**) 40ET.

The TEM examinations showed that the mean diameter of silica samples was mean 20 nm; they are highly monodispersed, nearly spherical and have a smooth surface (see Fig. 1). In the case of HS high negative zeta potential is expected, and the measurements confirm it. High number of free silanol groups at the surface causes the negative zeta potential, as their pK value is approximatively 4.5, which means that some of them are dissociated in the water suspension. The surface modification will decrease the number of free silanol groups on the surface, which should cause the decrease of the zeta potential under the same conditions. The zeta potential of HS suspended in water was ζ = −116 mV. After surface modification the zeta potential values decreased with increasing surface coverage. The values were ζ = −79 mV for 20ET and ζ = −63 mV for 40ET.

**Figure 2:**
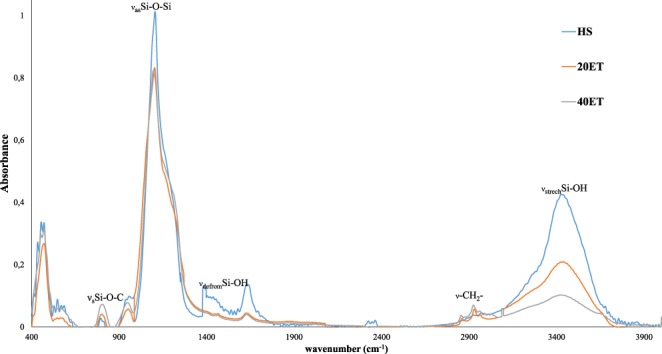
FT-IR spectra of silica nanoparticles.

The surface modification of SNPs was examined with FT IR spectroscopy. No significant difference in the position of the vibrational frequency for the SiO_2_ functional groups was observed (see Fig. 2). The surface modification of HS with ethyl functional groups caused the decrease of the number of Si-OH groups on the surface of SNPs, hence intensity of peaks belonging to ν_as_Si-O-Si (1105 cm^−1^), ν_defrom_Si-OH (1395 and 1645 cm^−1^) and ν_s__trech_Si-OH (3095–3685 cm^−1^) decreased, while the intensity of peaks of ν_s_Si-O-C increased (815 cm^−1^). New peak attributed to the ν-CH_2_- have appeared for the surface modified samples. Their vibrational frequencies are the same in both cases, 2855 and 2930 cm^−1^, but intensities are higher for the sample 40ET. The results clearly show that the surface modification was successful, and the intensity of peaks ν_s_Si-O-C and ν-CH_2_ correlate to the surface coverage with ethyl functional groups.

### GC-MS and GC-FID analysis of essential oils

The exact composition of EOs was determined with gas chromatography. The components were identified by comparing their retention times and relative retention factors with standards and oils of known composition. Two parallel measurements have been performed. The main components are the follows eugenol 78.64% in clove EO, cinnamaldehyde 63.77% in cinnamon bark EO, menthol 50.4% and menthon 19.8% in peppermint EO, thymol 39.88% and p-cymene 19.2% in thyme EO. In Table 1. other major components can be seen. We did not indicate or identify the compounds which were present significantly under 1%; this is the reason why the totals are not 100%.

**Table 1:** Composition of essential oils.

Results of GC measurements of essential oils					
*Component*	*RI*	*Percentage of compounds (%)*			
		*Peppermint EO*	*Thyme EO*	*Clove EO*	*Cinnamon bark EO*
*α*-Pinene	939	1.1	1.0	—	5.1
Camphene	951	—	2.0	—	-
*β*-Myrcene	992	—	1.0	—	—
Carvacrol		—	5.9	—	—
*α*-Terpinene	1017	—	3.2	—	—
*p*-Cymene	1026	—	19.2	—	1.9
Limonene	1044	1.4	—	—	1.8
1,8-Cineole	1046	5.5	4.6	—	2.8
*γ*-Terpinene	1060	—	6.7	—	—
Linalool	1104	—	5.6	—	4.0
Isopulegol	1150	1.0	—	—	—
Menthone	1156	19.8	—	—	—
Isomenthone	1159	7.0	—	—	—
Menthol	1172	50.4	—	—	—
Isomenthol	1183	4.3	—	—	—
*α*-Terpineol	1190	—	1.0	—	2.2
Pulegone	1215	1.9	—	—	—
*trans*-Cinnamaldehyde	1266	—	—	—	63.7
Bornyl acetate	1289	—	1.0	—	—
Thymol	1297	—	39.8	—	—
Isomenthyl acetate	1305	5.5	—	—	—
Eugenol	1373	—	—	78.8	4.6
*β*-Elemene	1394	—	—	—	—
*β*-Caryophyllene	1417	1.3	4.2	13.5	4.2
Cinnamyl acetate	1446	—	—	—	9.4
*α*-Humulene	1452	—	—	4.6	—
*β*-Cadinene	1473	—	—	1.1	—
**Total:**		**99.2**	**98.2**	**98.0**	**99.7**

The results of GC analysis show the average per cent of the two parallel measurements of volatile compounds in every case. The values of standard deviation were below 4.5%. RI: retention indices relative to C8–C30 n-alkanes. We did not indicate the unknown compounds and compounds under 1%.

### Preparation and characterisation of pickering nano-emulsions

The maximum concentration of EOs was set to MIC value for all examined emulsions. The MIC values of pure EOs in ethanolic solutions were previously determined against *Streptococcus mutans* with broth macrodilution test (see Part 3.4), for different EOs these were: clove EO 1.02 g/L, cinnamon EO 0.80 g/L, peppermint EO 1.96 g/L, thyme EO 0.40 g/L. We have prepared PnEs with HS, 20ET or 40ET stabilising agents, for CEs Tween80 surfactant was used, the concentrations of stabilising agents were 1 g/L for all experiments. The emulsions were stored at room temperature; t = 25 °C. Each experiment was made in triplicates. Stability of emulsions was determined from periodical droplet size determination with DLS (see Table 2.). The emulsions were considered to stable when the droplet size did not change within 24 hours, and creaming, sedimentation or disproportionation did not occur. Because of the large number of experimental data, we did not indicate data for the standard deviations of droplet size in Table 2. All values were in 1.2–8.3% range.

**Table 2:** Composition and characterisation of emulsions.

Parameters of Pickering- and conventional emulsions of essential oils				
*Essential oil*	*c*_*oil*_ *(g/L)*	*Stabilizing agent*	*D*_*droplet*_ *(nm)*	*Stability*
Clove EO	0.05–1.02	20ET	155–1660	2 weeks
	0.05–0.7	Tween80	155–245	2 weeks
	0.8–1.02	Tween80	335–455	1 weeks
Cinnamon EO	0.03–0.8	HS	400–4880	4 days
	0.03–0.8	20ET	185–280	2 months
	0.03–0.8	40ET	315–550	2 months
	0.03–0.5	Tween80	240–265	3 weeks
	0.6–0.8	Tween80	275–3010	2 weeks
Peppermint EO	0.11–1.96	20ET	210–11450	4 month
	0.105–0.7	Tween80	255–310	1 weeks
	0.8–1.96	Tween80	350–1090	2 weeks
Thyme EO	0.05–0.4	20ET	155–395	4 months
	0.05–0.4	Tween80	150–240	1 month

Droplet size and stability were calculated from data of 3 parallel samples. The concentration of stabilizing agent was constant 1 g/L. Droplet size standard deviation = ±1.2–8.3%. All emulsions are O/W type, which was determined with conductivity tests.

Because the volume fraction of EO (Θ_o_) was under 0.01 in every case, we could assume, that all PnEs and CEs were O/W type emulsions. We have performed filter paper tests with CoCl_2_ and dye test with Sudan red G to confirm this assumption^29^.

The results confirmed that the PnEs could have same or better stability than CEs (see Table 2), because of the high adsorption energy of solid nanoparticles on the liquid-liquid interface. The most stable emulsions are the thyme EO containing ones, in this case the stability of PnEs was 4 months, while its CEs were stable for only 1 month.

### Biofilm formation and treatment

The EOs concentration was MIC/2 value in all examined emulsions or solutions, which is a standard concentration for biofilm inhibition tests^32^. Our results of the crystal violet assay showed that the absolute ethanol and Tween80 solution have antibacterial effect, and they reduced the biofilm mass, while the HS, 20ET and 40ET nanoparticle suspensions had no antibacterial effect, and they did not reduce the biomass significantly.

**Figure 3:**
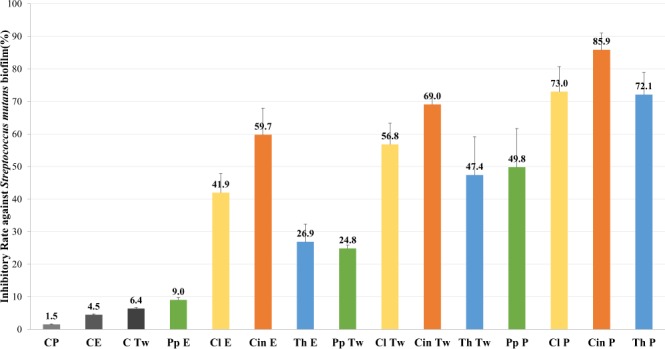
Results of biofilm inhibition tests. Biofilm inhibition activity of different formulated EOs against *Streptococcus mutans*, the concentration of EOs were the MIC/2 values. C: control. P: PnE form. E: ethanol/ethanolic solution. Tw: Tween80 solution/CEs with Tween80 surfactant. Pp: peppermint EO, MIC/2: 0.98 g/L. Cl: clove EO, MIC/2: 0.51 g/L. Cin: cinnamon EO, MIC/2: 0.40 g/L. Th: thyme EO, MIC/2: 0.20 g/L.

Even so, the PnEs prepared with 20ET were the most effective forms in biofilm inhibition. For each EOs, the ethanolic solutions and CEs showed less biofilm inhibition (see Fig. 3.); e.g. in the case of thyme EO, the inhibitory rates (IR) were 26.9, 47.4 and 72.1% for ethanolic solution, CE and PnE respectively.

**Figure 4:**
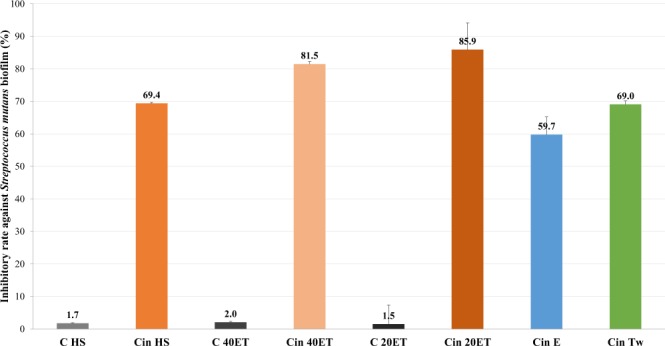
Results of biofilm inhibition tests in case of cinnamon EO. Biofilm inhibition activity of different formulated cinnamon EO against *Streptococcus mutans*. The concentration of cinnamon EO was its MIC/2 values, 0.40 g/L. C: control. HS: PnE with HS stabilising agent. 20ET: PnE with 20ET stabilising agent. 40ET: PnE with 40ET stabilising agent. E: ethanol/ethanolic solution. Tw: CE with Tween80 surfactant.

We have found cinnamon EO have the highest inhibition effect among tested EOs. The inhibitory rates for its different forms are 59.7 for ethanolic solution, 69.0% for CE and 85.9% for PnE respectively. In this case we have performed tests on the influence of the hydrophilic/lipophilic surface properties of stabilising SNPs on the inhibitory effect on PnE. HS and 40ET nanoparticles stabilised PnEs were also tested in biofilm inhibition. The results showed (Fig. 4), that in the case of 20ET nanoparticles stabilised PnE had better biofilm inhibition effect (IR 85.9%) than 40ET (IR 81.5%), and HS stabilised ones (IR 69.4%).

**Figure 5:**
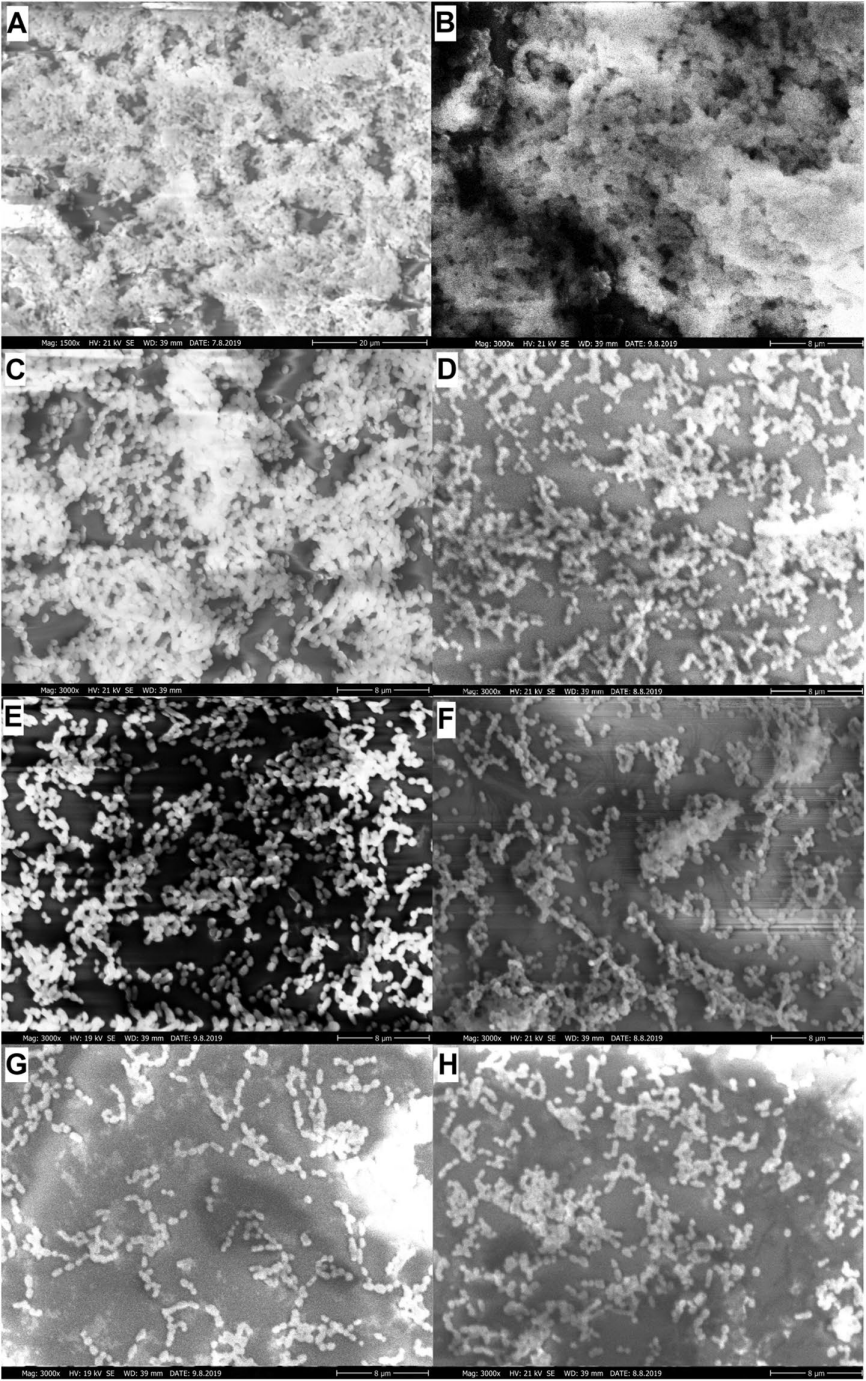
SEM images of biofilm and biofilms after treatment with a different formulation of cinnamon EO. The EO concentrations equal with the MIC/2. (**A**,**B**) Control *Streptococcus mutans* biofilm, untreated. (**C**) Biofilm treated with PnE of peppermint EO. (**D**) Biofilm treated with PnE of clove EO. (**E**) Biofilm treated with PnE of thyme EO. (**F**) Biofilm treated with HS stabilised PnE of cinnamon EO. (**G**) Biofilm treated with 20ET stabilised PnE of cinnamon EO. (**H**) Biofilm treated with 40ET stabilised PnE of cinnamon EO. The magnification is 1500x and 3000x for biofilm control, and 3000x for other samples, scale bar is 8 µm.

In Fig. 5 we can see the SEM images of untreated and treated biofilms. Figure 5A,B are the images of untreated control biofilms. The *in vitro* adherence of *Streptococcus mutans* colonies can be clearly seen, and thick coherent biofilm has formed on the surface. The SEM images bear out the results of biofilm inhibition experiments. The PnE of peppermint EO reduced the biofilm mass (Fig. 5C) minimally, while in the images of biofilms treated with PnE of clove (Fig. 5D) and thyme EO (Fig. 5E) we can see bacterial colonies on the surface, but the adherence of coherent biofilm was reduced.

The SEM images clearly show, high inhibitory effect of the PnEs of cinnamon EO. On the images of biofilms treated with HS (Fig. F) and 40ET (Fig. H) stabilized PnEs of cinnamon EO we can see some coherent biofilm spots, while on the image of biofilm treated with 20ET stabilized PnE of cinnamon EO (Fig. G), no coherent biofilm formation can be observed.

In all the cases, a very good correlation of the inhibitory rate obtained by the crystal violet assay and the data obtained from SEM images can be found.

### *In vitro* diffusion studies

**Figure 6:**
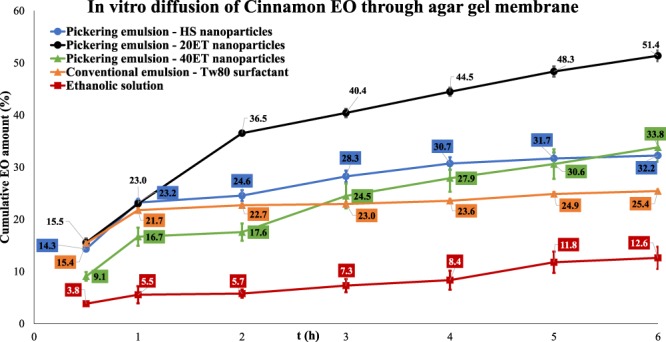
*In vitro* diffusion of cinnamon EO through agar gel membrane. The cinnamon EO concentration is 0.40 g/l (MIC/2 against Streptococcus mutans). The stabilising agent of PnEs are HS, 20ET, and 40ET nanoparticles, the stabilising agent of CE is Tween80. The concentration of emulsion stabilising agents is 1 g/l. The droplet size of CE is D = 320 nm, the PnE with HS D = 220, PnE with 20ET D = 245 nm, and PnE with 40ET D = 255.

After the discussion on the results obtained from biofilm inhibition tests, an assumption has been made that there should be a correlation between inhibitory rate and diffusion properties trough biofilm of the EOs in different forms. To confirm this assumption, *in vitro* diffusion tests were performed. Static Franz diffusion cell method was used with agar gel as model membrane. The EOs concentration was the same that was used for the biofilm inhibition tests, MIC/2 values. The droplet size of the different types of emulsions was similar (see Table 3), and we could assume that the diffusion properties depend only on the type of the emulsion or surface properties of emulsion stabilising agent. The diffusion profiles of different EOs are very similar; this is why we have graphically presented only the *in vitro* diffusion study curve of cinnamon EO as it has shown the highest inhibitory rate (see Fig. 6). The results of cumulative amount after 6 hours for all samples can be seen in Table 3. The cumulative amount of EO means the diffused EO amount after 6 hours. The diffusion curves of all the other samples can be seen in Supplementary Information.

**Table 3:** Results of *in vitro* diffusion studies.

Results of *in vitro* diffusion studies				
*Essential oil*	*c*_*oil*_ *g/l*	*Formula*	*Droplet size D (nm)*	*Cumulative EO release(%)*
Clove	0.51	Ethanolic solution	—	12.9 ± 2.8
		Conventional emulsion	320 ± 37	21.5 ± 0.1
		Pickering nano-emulsion	370 ± 22	27.5 ± 4.0
Cinnamon	0.40	Ethanolic solution	—	10.5 ± 1.6
		Conventional emulsion	240 ± 20	30.7 ± 1.2
		Pickering nano-emulsion HS	220 ± 4	32.3 ± 1.2
		Pickering nano-emulsion 20ET	245 ± 12	51.4 ± 1.0
		Pickering nano-emulsion 40ET	255 ± 6	33.8 ± 2.5
Peppermint	0.98	Ethanolic solution	—	69.7 ± 14.1
		Conventional emulsion	310 ± 9	69.0 ± 9.2
		Pickering nano-emulsion	210 ± 10	81.1 ± 2.2
Thyme	0.20	Ethanolic solution	—	under LOD
		Conventional emulsion	245 ± 20	9.4 ± 0.4
		Pickering nano-emulsion	255 ± 5	18.9 ± 0.5

The oil concentrations equal to MIC/2 values against Streptococcus mutans. Droplet sizes and cumulative EO releases were calculated from three parallel samples.

Generally, we can conclude that the cumulative EO amounts were highest for PnEs. In the case of cinnamon EO these values are: 51.4% for 20ET stabilised PnE, 30.7% for CE and 10.5% for ethanolic solution respectively. At thyme EO, cumulative EO amount for ethanolic solution was under the limit of detection value.

### Conclusions

We have successfully prepared O/W type PnEs of EOs with droplet size tailored for diffusion trough biofilm porous structure (210–370 nm). For the stabilisation of PnEs, we have synthesised and surface-modified spherical silica nanoparticles. These emulsions remained stable for at least 1 week, in some cases more, then 4 months.

We have determined MIC values against *Streptococcus mutans* for all examined EOs, and we have performed a biofilm inhibition tests applying EOs in MIC/2 amount. We have tested tree forms for all examined EOs, ethanolic solution, CE and PnE. The test results shown that the PnE form has highest antimicrobial effectiveness compared to CE or ethanolic solutions for all examined EOs. We assume that the difference in effect should correlate with the penetration of EOs in different form trough polysaccharide biofilm structure. To confirm this idea we have studied the diffusion trough model membrane (agar gel). The results have shown that for all samples, there is a direct relation of antimicrobial or inhibitory effect with diffusion properties of EOs in different form. The PnE form had the highest cumulative amount for all EOs, and ethanolic solution had the lowest cumulative amount. This result is an explanation why the PnE have antibacterial effect while ethanolic solution with the same EO concentration have inhibitory effect, which is clearly seen on the SEM images. The highest difference in the inhibitory rate of different forms has been observed in the case of thyme oil. The thyme oil has the highest antimicrobial effectiveness in comparison with other examined EOs as its MIC value is lowest. The ethanolic solution did not provide a measurable amount of thyme EO trough model membrane, and it had a very low inhibitory rate (27%). The CE form had a cumulative amount of 9%, and the inhibition rate has increased to 47%, while the PnE form had 19% of cumulative amount, and the inhibition rate increased to 72%. These results indicate that the delivered amount of an EO with high antimicrobial effectiveness to the biofilm has the greatest effect on the inhibition activity. Beside the droplet size, the appropriate hydrophilic/lipophilic surface character is very important for effective delivery of EO to biofilm. We have found that PnEs stabilised with silica that has a moderate hydrophobic character (20ET), shows the highest delivery of EOs to biofilm that is accompanied with highest inhibitory effect.

We can conclude that O/W type PnEs stabilised by nanoparticles with appropriate hydrophilic/hydrophobic surface properties, provide a new possibility for the application of EOs in pharmaceutical treatment against *Streptococcus mutans* biofilm formation.

## Materials and Methods

### Materials

Chemicals for experiments were all analytical grade and used as received. The sources and grade of all chemicals and materials used for experiments are discussed in the Supplementary Information.

### Synthesis- surface modification and characterisation of silica nanoparticles

Synthesis of hydrophilic silica was performed based on the work of Stöber, Fink, and Bohn^33^. The optimisation of the synthesis process and surface modification with ETES was performed in our previous work^31^. We characterised the silica nanoparticles with DLS, TEM and FT-IR measurements. The brief synthesis route and further information about the measurements are given in the Supplementary Information.

### GC-MS and GC-FID conditions

Detailed information on the applied analytical method can be read in the Supplementary Information.

### Broth macrodilution test (BDT)

With this test, we determined the MIC and MIC/2 values of each EO against *Streptococcus mutans*. The test was based on the recommendations of the Manual of Clinical Microbiology^34^ associated with modifications published before^35^. Further details are given in the Supplementary Information.

### Preparation and characterisation of O/W type PnEs

The concentration of emulsifiers was set to 1 mg/ml and was kept constant for all experiments. The influence of EOs concentration on the emulsion droplet size was examined; it was varied until the minimum inhibitory concentration against *Streptococcus mutans* (see Table 2.) The exact emulsification process, the droplet size, and stability measurements can be seen in the Supplementary Information.

### *In vitro* diffusion studies – Static Franz Diffusion cell method

The examination of diffusion properties was performed in static vertical Franz diffusion cells (Hanson Microette Plus. Hanson Research 60-301-106). The essential oil content of samples was determined with UV-Vis spectroscopy (Jasco V-550 UV/VIS Spectrophotometer). To compare the effectiveness of PnEs, we examined the diffusion of EOs in an ethanolic solution and emulsion stabilised with Tween80 surfactant. Further information and details about the *in vitro* diffusion study are given in the Supplementary Information.

### Biofilm inhibition experiments

The biofilm inhibition experiments were performed on the base of Peeters and co-worker’s study, with the crystal violet assay^36^. The detailed method is written in the Supplementary Information.

### Preparation of the biofilm samples for Scanning Electron Microscopy

The biofilm was imaged by SEM (JEOL JSM-6300) as previously described^37^ with some modification (briefly see Supplementary Information). To determine the effect of EOs and different formulations, we have used control samples that were treated with ethanol and Tween80 solutions as well with the suspension of HS, 20ET or 40ET nanoparticles. Their concentration was the same as it was in the ethanolic solution, CEs, and PnEs^38^.

## Supplementary information

Supplementary Information
