# Supplementary material for: Preparation, characterisation and microbiological examination of Pickering nano-emulsions containing essential oils, and their effect on Streptococcus mutans biofilm treatment
Source: Sci Rep. 2019 Nov 12;9:16611. doi: 10.1038/s41598-019-52998-6 (PMC6851100; doi:10.1038/s41598-019-52998-6)
Supplement: Supplementary file 1 — Supplementary Information [file 41598_2019_52998_MOESM1_ESM.docx]

*Preparation, characterisation and microbiological examination of Pickering nano-emulsions containing essential oils, and their effect on Streptococcus mutans biofilm treatment*

Barbara Horváth^1^#, Viktória L. Balázs^2^#, Adorján Varga^3^, Andrea. Böszörményi^4^, Béla Kocsis^3^, Györgyi Horváth^2^, Aleksandar Széchenyi^1*^

^1^Institue of Pharmaceutical Technology and Biopharmacy, Faculty of Pharmacy, University of Pécs. Rókus str. 2., H-7624 Pécs, Hungary

^2^Department of Pharmacognosy, Faculty of Pharmacy, University of Pécs, Rókus str. 2., H-7624 Pécs, Hungary

^3^Department of Medical Microbiology and Immunology, Faculty of Medicine, University of Pécs, Szigeti str, 12., H-7624 Pécs, Hungary

^4^Department of Pharmacognosy, Faculty of Pharmacy Semmelweis University, Üllői str, 26., H-1085 Budapest, Hungary

#First authors with equal contribution

*corresponding author, [szealex@gammat.ttk.pte.hu](mailto:szealex@gammat.ttk.pte.hu), +3672/501500/28801

**Chemicals and materials**

For the synthesis and surface modification of silica nanoparticles, the following chemicals were used: tetraethoxysilane [TEOS]. (Alfa Aesar [USA]. 98%). ethyltriethoxysilane [ETES] (Alfa Aesar [USA]. 96%). absolute ethanol (VWR Chemicals [Hungary]. AnalaR Normapur. ≥99.8%). 28 w/w% ammonium solution (VWR Chemicals [Hungary]. AnalaR Normapur. analytical reagent).

Clove-. cinnamon-. peppermint- and thyme EOs were used, all of them were purchased from Aromax Ltd. (Hungary). The main components of the EOs (eugenol. cinnamaldehyde. menthol and thymol) were bought from Sigma-Aldrich (Budapest. Hungary). The stabilising agent of conventional emulsions was Tween^®^80 (Polysorbate80 Acros Organics. USA).

Na_2_HPO_4_ (VWR Chemicals [Hungary], anhydrous≥99%. AnalaR Normapur. Reag. Ph. Eur.). KH_2_PO_4_ (VWR Chemicals [Hungary], AnalaR Normapur. Reag. Ph. Eur.), NaCl (VWR Chemicals [Hungary] AlanaR Normapur. Reag. Ph. Eur.), KCl (VWR Chemical [Hungary]. AnalaR Normapur. Reag. Ph. Eur.), (VWR Chemicals [Hungary]. 37w/w% Ph. Eur.), ultrapure water (membraPure Astacus. Analytical with UV) were used for PBS buffer preparation. Agar gel membranes have been prepared as described before[1], before the diffusion experiments it was cut into the 38 mm diameter and 2.1 mm thick disks and placed into the membrane holder of the vertical diffusion cells.

For the microbiological experiments, the following ingredients were used: *Streptococcus mutans* standard (Leibniz Institut DSMZ GmbH. DSMZ 20533), Tween^®^80 (Polysorbate80 Acros Organics. USA)., Brain Heart Infusion [BHI]. (Sigma-Aldrich. Hungary), sheep blood agar (5 V/V% defibrinated blood), BRAND®plates. 96 pureGrade S PS Trans F-B (VWR Chemicals. Hungary), absolute ethanol (Molar Chemicals. Hungary. analytical reagent), crystal violet 1% aqueous solution. (Sigma-Aldrich. Hungary. analytical reagent), 33 w/w% acetic acid (Molar Chemicals. Hungary. purity 99-100%).

For the SEM sample preparation we have used: absolute ethanol (Molar Chemicals. Hungary), glutaraldehyde (Molar Chemicals. Hungary. purum), acetone (Molar Chemicals. Hungary. analytical reagent), terc-butanol (Molar Chemicals. Hungary. analytical reagent).

**Synthesis-, surface modification and characterisation of silica nanoparticles**

Synthesis of hydrophilic silica was performed based on the work of Stöber, Fink and Bohn. The optimisation of the synthesis process and surface modification with ETES was performed in our previous work. In brief, the mixture of water/ethanol/TEOS/NH_3_ with molar ratio 100:300:5.2:1 was stirred on room temperature, 25°C for 24 h, followed by the addition of 0.023 or 0.046 mole fraction of ETES and stirred for additional 6 h at the same temperature. The synthesised silica nanoparticles were stored and found to be stable without aggregation and any change in size or morphology for several months in the residual reaction solution. Before using the ammonium hydroxide and ethanol content were always removed by distillation (Heidolph. Laborota 4000), from modified silica suspension. The water content was supplemented three times. The concentration of silica nanoparticle water-based suspension was finally adjusted to 1 mg/cm^3^.

The size distribution was determined by dynamic light scattering (DLS) using a Malvern Zetasizer Nano S instrument. The zeta potential of silica nanoparticles was determined by DLS using a Malvern Zetasizer Nano Z instrument. The size distribution was confirmed, and morphology of silica nanoparticles was studied with Transmission Electron Microscopy (TEM), JEM 1200 EX II and JEOL-1400 TEM. For TEM experiments 200 mesh copper grids (Micro to Nano Ltd.) were coated with Butvar B-98 thin film, and the samples were dropped cast on the Butvar film from a tenfold diluted suspension. FTIR spectra of the silica nanoparticles (~1 mg) were recorded with a single beam Thermo Nicolet 5700 FT-IR spectrophotometer (Thermo Electron Corporation, USA), with the following scan parameters: scan range 4000–400 cm^-1^; number of scans 32; resolution 4 cm^-1^; interval 1.0 cm^-1^. The sample preparation for FT IR measurements was the follows: the appropriate amount of silica nanoparticles suspension was centrifuged at 15 000 rpm for 45 min, and washed with absolute ethanol three times; then the SNPs were dried at 105°C for 24 hours; finally KBr pastilles with SNPs were prepared.

**GC-MS and GC-FID measurements of essential oils**

1 µL of essential oil samples diluted in ethanol (10µL/mL) was injected in split mode, the injector temperature was 250°C and the split ratio was 1:50. The analyses were carried out with an Agilent 6890N/5973N GC-MSD (Santa Clara. CA. USA) system equipped with an Supelco (Sygma-Aldrich) SLB-5MS capillary column (30 m × 250 µm × 0.25 µm) for GC-MS measurements and a J&W (Agilent) DB-5MS capillary column (25 m × 250 µm × 0.25 µm) for GC-FID measurements. The GC oven temperature was programmed to increase from 60ºC (3 min isothermal) –250ºC at 8 ºC/min (1 min isothermal). High purity helium (6.0) was used as carrier gas at 1.0 mL/min (37 cm/s) in constant flow mode.

The mass selective detector (MSD) was equipped with a quadrupole mass analyser and was operated in electron ionisation mode at 70 eV in full scan mode (41–500 amu at 3.2 scan/s). The temperature of the flame ionisation detector (FID) was 300 ºC. He flow was 30 mL/min; airflow was 400.0 mL/min. the makeup gas was Nitrogen in constant flow mode (25 mL/min).

The data were evaluated using MSD ChemStation D.02.00.275 software (Agilent). The identification of the compounds was carried out by comparing retention times and recorded spectra with the data of authentic standards, and the NIST 2.0 library was also consulted. The percentage of the evaluation was carried out by area normalisation.

**Broth macrodilution test (BDT)**

From each EOs 5 mg/ml stock solutions were made using ethanol as the solubilising agent, the concentration of surfactant was 0.1 w/w%, the solvent was BHI medium. Conventional emulsions and Pickering nanoemulsions of EOs samples of EOs in 5 mg/ml concentration were also prepared for BDT test. From Streptococcus mutans bacterial culture 1 ml (~ 4 × 107 cells/mL) was added to1 ml 5 mg/ml EO stock solution in BHI medium, a serial twofold dilution was prepared from 5 to 0.0391 mg/mL, and each tube was incubated at 37°C for 24 h. As control of the bacterial growth, neither EO nor detergent was added to the tubes. After the incubation time, 5 w/w% sheep blood agar were prepared, and the content of every tube was inoculated. The Petri dishes were incubated at 37°C for 24h. The number of bacterial colonies was compared to the controls and the values of the minimum inhibitory concentrations (MIC) were determined. The MIC value is the concentration that could reduce the visible growth of bacteria in comparison with the controls. All tests were carried out in triplicates.

| **Essential oil** | **Form** | **MIC (g/L)** | **MIC/2 (g/L)** |
| --- | --- | --- | --- |
| Clove EO | Ethanolic solution | 1.02 | 0.51 |
|  | CE | 1.00 | 0.50 |
|  | PnE | 0.5 | 0.25 |
| Cinnamon EO | Ethanolic solution | 0.80 | 0.40 |
|  | CE | 0.70 | 0.35 |
|  | PnE with HS | 0.25 | 0.125 |
|  | PnE with 20ET | 0.125 | 0.063 |
|  | PnE with 40ET | 0.125 | 0.063 |
| Peppermint EO | Ethanolic solution | 1.96 | 0.98 |
|  | CE | 1.55 | 0.775 |
|  | PnE | 0.50 | 0.25 |
| Thyme EO | Ethanolic solution | 0.40 | 0.20 |
|  | CE | 0.40 | 0.20 |
|  | PnE | 0.25 | 0.125 |

1. Table: Results of broth macrodilution test.

**Preparation and characterisation of O/W type EOs Pickering nano-emulsions**

The concentration of emulsifiers was set to 1 mg/ml and was kept constant for all experiments. The influence of EOs concentration on the emulsion droplet size was examined; it was varied until the minimum inhibitory concentration against Streptococcus mutans was reached (see Table 2.) The emulsification was performed in two steps. In the pre-emulsification process, the mixtures were sonicated for 2 minutes (Bandelin Sonorex RK 52H). The final emulsification was performed with UltraTurrax (IKA Werke T-25 basic) for 2 minutes at 13 500 rpm. The emulsions droplet size was determined with DLS using a Malvern Zetaziser Nano S instrument. Stability of the emulsions was determined from periodical droplet size determination.

**Biofilm inhibition experiments**

The biofilms were prepared in 96 well microtiter plates, 200 µl bacterial culture (4x107 cells/ml) was added into each well, then microtiter plate was incubated for 4 hours at 37°C, to help the adhesion of the cells. After the incubation time, the non-adherent cells were washed with physiological saline solution. Ethanolic solutions, conventional emulsions with Tween80 surfactant and Pickering nano-emulsions of EOs were used for the experiments. The concentration of EOs were the MIC/2 values, which were determined with the broth macrodilution tests. As a positive control, we had used untreated sample, when only BHI medium was added to the bacterial culture.

Further controls without EOs included Tween80 solutions. HS, 20ET or 40ET nanoparticle suspensions, and ethanol. The concentration of solutions or suspensions were the same as well as the concentration of stabilising agents in the emulsions. After the treatment, the microtiter plate was incubated again for 24 hours at 37°C. The adherent cells were fixed with methanol for 15 min. The biofilms were stained with 0.1 % crystal violet solution for 20 min. The redundant dye was washed with distilled water, 150 µl 33 w/w% acetic acid was added to each plate, and the absorbance was measured at λ= 595 nm (BMG Labtech SPECTROstar Nano). The crystal violet reacts with polysaccharide matrix of biofilm and with the extracellular matrix of the biofilm, so the amount of biomass can be determined with spectroscopic method.

**Preparation of the biofilm samples for Scanning Electron Microscopy**

The biofilms were prepared on microscope cover glasses 24×32 mm by incubation in 5 mL bacterial culture [4 × 107cells/ml] with 4 hours incubation time at 37°C. After the first incubation, the samples were washed with physiological saline solution and treated with EOs in MIC/2 concentrations in the form of ethanolic solutions, conventional emulsions, and Pickering nano-emulsions. To determine the effect of EOs and different formulations, we have used control samples that were treated with ethanol and Tween80 solutions as well with the suspension of HS. 20ET or 40ET nanoparticles. Their concentration was the same as it was in the formulations. The incubation time was 24 hours at 37°C, followed by washing with physiological saline solution. The preparation of the samples for electron microscopy was done as described by Kerekes et al , the protocol is briefly the following: the samples were soaked with 2.5% glutaraldehyde in 0.05 M cacodylate buffer (pH=7.5), for 2 h at room temperature; then dehydrated with different ethanol concentrations: 50%, 70%, 80%, 90%, 95% and 98%. Each ethanol treatment lasted for 2x15 min at room temperature. The dehydration procedure was continued with alcoholic treatment: the samples were treated with a mixture of terc-butanol and absolute ethanol, the volumetric ratio was 1:2. 1:1. 2:1, and in the last step, pure terc-butanol, for 5 min. Then the samples were frozen in a so-called pre-freeze process with terc.-butanol at -20°C, and they were lyophilised (Heto Drywinner DW 1.0-60E) at -40°C and 4·10-4 mbar for 8 hours. Finally, the samples were coated with gold and examined with JEOL JSM-6300 Scanning Electron Microscope.

The SEM images about biofilms treated with ethanolic solution of cinnamon EO (A) and with the conventional emulsion of cinnamon EO can be seen in Fig. 1.


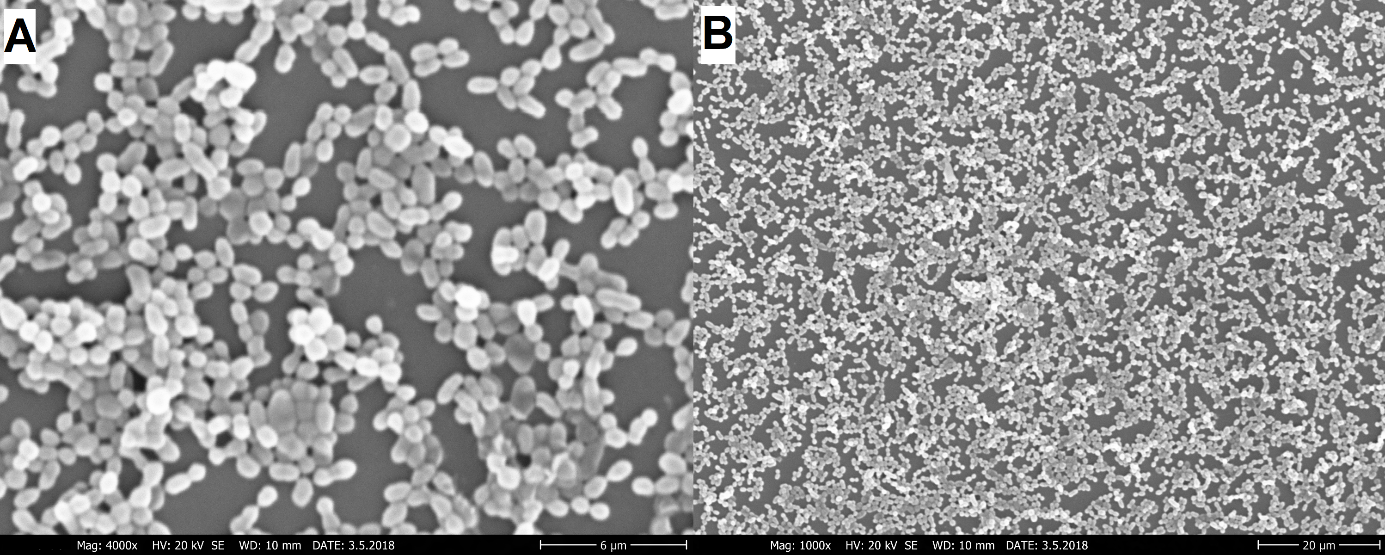


1. Figure: SEM images of biofilms after treatment with different formulation of cinnamon EO. The EO concentration is equal with the MIC/2: 0.40 g/L. **A:** Streptococcus mutans biofilm treated with an ethanolic solution. **B:** Streptococcus mutans biofilm treated with conventional, Tween80 stabilised emulsion.

**In vitro diffusion studies – Static Franz Diffusion cell method**

The examination of diffusion properties was performed at 37°C in static vertical Franz diffusion cells (Hanson Microette Plus. Hanson Research 60-301-106), six parallel cells were used with 2.1 mm thick, two w/w% agar gel membranes with effective penetration area of 2.54 cm2. The volume of the receiver chamber was 7 ml, and the receiver solution was PBS buffer. The volume of 600 μl for emulsion or solution sample was placed in the donor chamber, and the diffusion was examined for 6 hours. In the first hour, 2 ml of sample was collected every 30 minutes, and later, the samples were taken every 60 minutes. The withdrawn sample volume was replaced with fresh PBS buffer. The essential oil content was determined with UV-Vis spectroscopy (Jasco V-550 UV/VIS Spectrophotometer).

The wavelength of absorption maximum are the follows clove EO λ_max_=281 nm, cinnamon EO λ_max_=292 nm, peppermint EO λ_max_ =237 nm, thyme EO λ_max_=277 nm. The Tween80 surfactant has an absorption maximum at 209 nm wavelength, and the silica nanoparticles have an absorption peak at 210 nm. The peppermint oil and emulsion stabilising agents have overlapping absorbance; for this reason, their concentration was determined by the application of multi-wavelength linear regression method. The concentration of EOs were MIC/2 values. 0.98 mg/ml for peppermint EO. 0.4 mg/ml for cinnamon bark EO. 0.51 mg/ml for clove EO and 0.20 mg/ml for thyme EO, which were determined with the broth macrodilution tests. To compare the effectiveness of Pickering nano-emulsions, we have examined the diffusion of EOs in an ethanolic solution and emulsion stabilised with Tween80 surfactant.

The results of in vitro diffusions studies of every EO samples can be seen in Fig. 2, 3, 4 and 5.


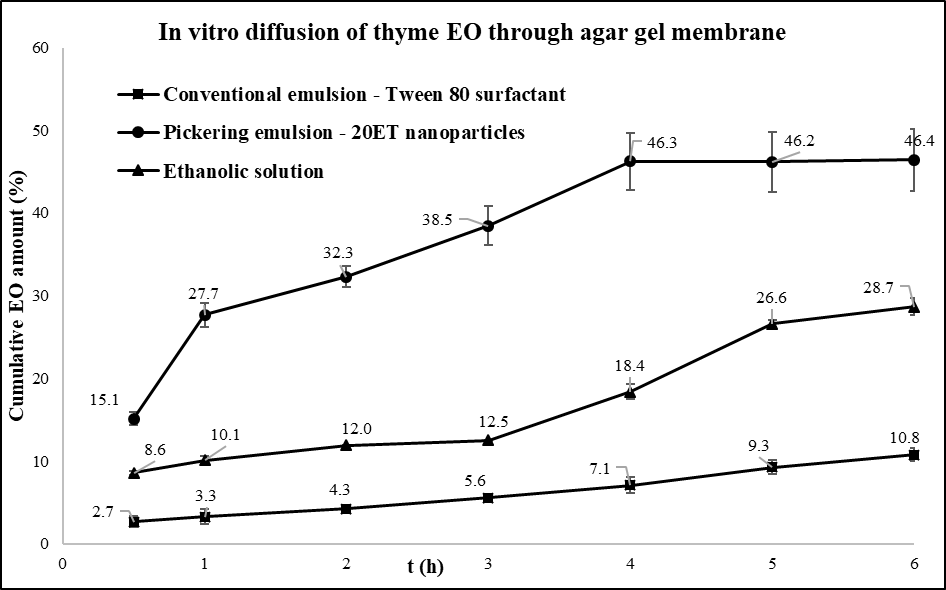


Fig. 2.: In vitro diffusion of thyme EO through agar gel membrane. The thyme EO concentration is 0.20 g/l (MIC/2 against Streptococcus mutans). The stabilising agent of PnE is 20ET nanoparticles. The stabilising agent of the conventional emulsion is Twenn80. The concentration of emulsion stabilising agents is 1 g/l. The droplet size of conventional emulsion is D=245.2 nm, the PnE D=254.0 nm.


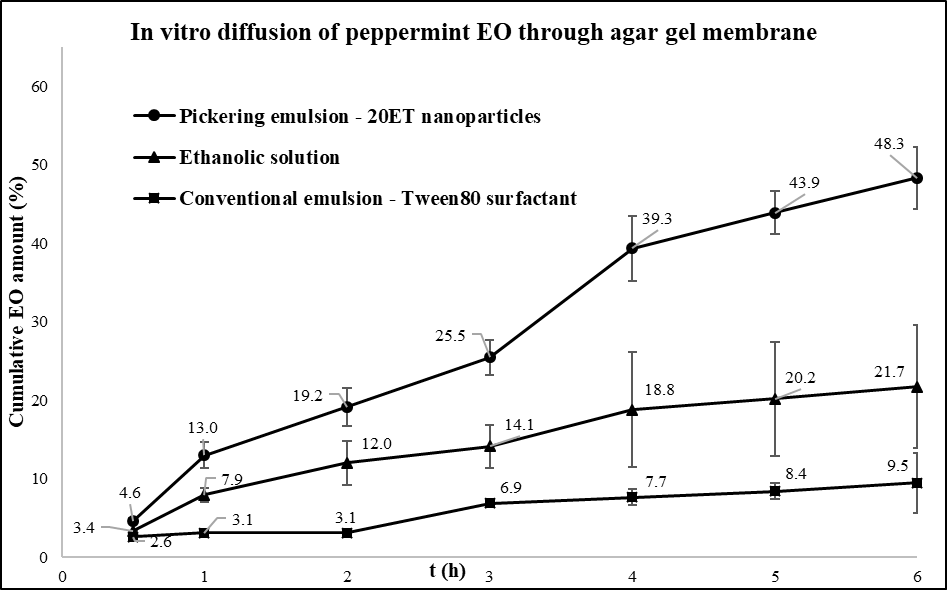


Fig. 3.: In vitro diffusion of peppermint EO through agar gel membrane. The peppermint EO concentration is 0.98 g/l (MIC/2 against Streptococcus mutans). The stabilising agent of PnE is 20ET nanoparticles. The stabilising agent of conventional emulsion is Twenn80. The concentration of emulsion stabilising agents is 1 g/l. The droplet size of conventional emulsion is D=212 nm, the PnE D=307 nm.


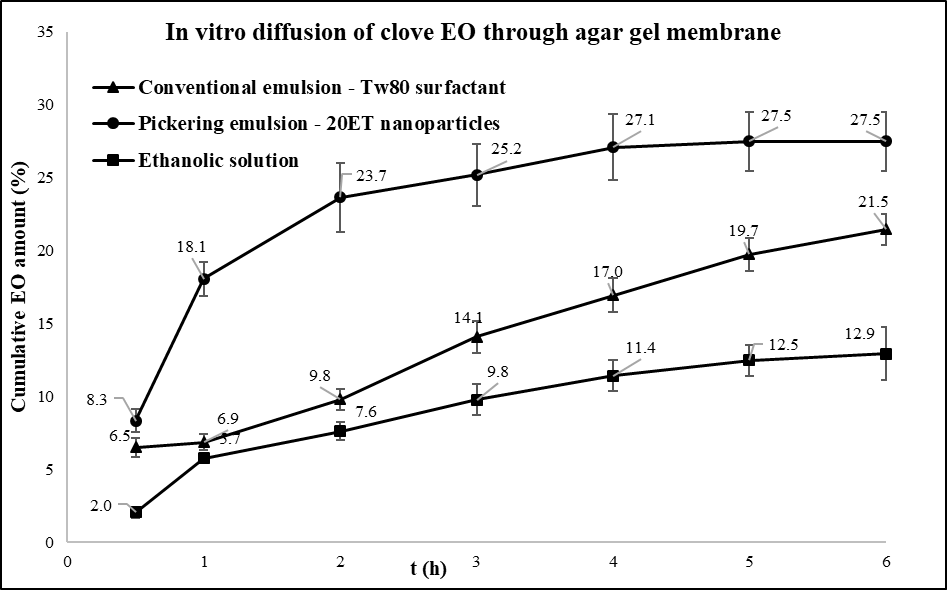


Fig. 4.: In vitro diffusion of clove EO through agar gel membrane. The clove EO concentration is 0.51 g/l (MIC/2 against Streptococcus mutans). The stabilising agent of PnE is 20ET nanoparticles. The stabilising agent of conventional emulsion is Tween80. The concentration of emulsion stabilising agents is 1 g/l. The droplet size of conventional emulsion is D=320 nm, the PnE D=372 nm.

[1] Sanders E.R. Aseptic Laboratory Techniques: Plating Methods. *J. Vis. Exp*. **63, e**3064 (2012)
